# Rare mutations and potentially damaging missense variants in genes encoding fibrillar collagens and proteins involved in their production are candidates for risk for preterm premature rupture of membranes

**DOI:** 10.1371/journal.pone.0174356

**Published:** 2017-03-27

**Authors:** Bhavi P. Modi, Maria E. Teves, Laurel N. Pearson, Hardik I. Parikh, Piya Chaemsaithong, Nihar U. Sheth, Timothy P. York, Roberto Romero, Jerome F. Strauss

**Affiliations:** 1 Department of Human and Molecular Genetics, Virginia Commonwealth University, Richmond, VA, United States of America; 2 Department of Obstetrics and Gynecology, Virginia Commonwealth University, Richmond, VA, United States of America; 3 Department of Anthropology, Pennsylvania State University, University Park, PA, United States of America; 4 Department of Microbiology and Immunology, Virginia Commonwealth University, Richmond, VA, United States of America; 5 Perinatology Research Branch, *Eunice Kennedy Shriver* National Institute for Child Health and Human Development, NIH, Bethesda, MD and Detroit, MI, United States of America; 6 Center for Study of Biological Complexity, Virginia Commonwealth University, Richmond, VA, United States of America; 7 Department of Obstetrics and Gynecology, University of Michigan, Ann Arbor, MI, United States of America; 8 Department of Epidemiology and Biostatistics, Michigan State University, East Lansing, MI, United States of America; 9 Center for Molecular Medicine and Genetics, Wayne State University, Detroit, MI, United States of America; Stellenbosch University, SOUTH AFRICA

## Abstract

Preterm premature rupture of membranes (PPROM) is the leading identifiable cause of preterm birth with ~ 40% of preterm births being associated with PPROM and occurs in 1% - 2% of all pregnancies. We hypothesized that multiple rare variants in fetal genes involved in extracellular matrix synthesis would associate with PPROM, based on the assumption that impaired elaboration of matrix proteins would reduce fetal membrane tensile strength, predisposing to unscheduled rupture. We performed whole exome sequencing (WES) on neonatal DNA derived from pregnancies complicated by PPROM (49 cases) and healthy term deliveries (20 controls) to identify candidate mutations/variants. Genotyping for selected variants from the WES study was carried out on an additional 188 PPROM cases and 175 controls. All mothers were self-reported African Americans, and a panel of ancestry informative markers was used to control for genetic ancestry in all genetic association tests. In support of the primary hypothesis, a statistically significant genetic burden (all samples combined, SKAT-O p-value = 0.0225) of damaging/potentially damaging rare variants was identified in the genes of interest—fibrillar collagen genes, which contribute to fetal membrane strength and integrity. These findings suggest that the fetal contribution to PPROM is polygenic, and driven by an increased burden of rare variants that may also contribute to the disparities in rates of preterm birth among African Americans.

## Introduction

Although there is strong evidence from twin-based studies that both maternal and fetal genetic factors contribute to preterm birth, attempts to identify specific loci contributing to prematurity in genome-wide association studies (GWAS) have largely failed to yield robust and reproducible findings [1–5]. A number of candidate gene association studies have found significant relationships, but meta-analyses indicate that these associations are at best weak or population-specific [6–8]. The disappointing output from genetic studies may relate to the subject inclusion and exclusion criteria, including the proximate cause of preterm birth—spontaneous preterm birth (sPTB) or preterm premature rupture of membranes (PPROM), population heterogeneity (partially attributable to genetic admixture), and environmental exposures, including viral and bacterial infections. Further, identification of genetic variants contributing to preterm birth risk is mostly based on the “common disease-common variant” hypothesis, which assumes that a large number of common allelic variants can explain the genetic variance in complex diseases. This approach has been employed for several complex traits such as human height and schizophrenia and despite having large sample sizes required for success, there still exists an issue of “missing heritability” with identified variants being able to explain only a small fraction of the genetic variance observed [9]. Considering the complex genetic nature of preterm birth, a systematic identification of even a small number of rare variants with moderate-large effect sizes in genes of known/putative biological significance can be helpful [3, 10]. None of the genetic studies on preterm birth so far have taken this approach.

PPROM is the leading identifiable cause of preterm birth, with ~40% of preterm deliveries being associated with PPROM [11]. Previous studies have focused on selected candidate genes involved in infection/ inflammation pathways, with the majority of these studies investigating only maternal genomes [8, 12–15] despite evidence of a fetal contribution established by twin studies [2, 4].

Human fetal membranes consist of an inner layer, the amnion, and an adherent outer layer, the chorion. The amnion is the load-bearing component of the fetal membranes and major contributor to their structural integrity. The strength of the fetal membranes is thought to be influenced by both synthesis and degradation of the extracellular matrix (ECM) components. Fibrillar collagens and associated proteins are major components of the fetal membranes contributing to their tensile strength [16,17]. Thus, defects in the fibrillar collagen synthesis and/or altered ECM metabolism can adversely affect fetal membrane integrity, and may result in preterm birth as a result of preterm rupture. Epidemiological studies show that fetuses/neonates with Ehlers-Danlos syndrome, osteogenesis imperfecta, and restrictive dermopathy, disorders of ECM synthesis, are at increased risk of adverse pregnancy outcomes including PPROM [17–20]. In addition, a functional promoter SNP in the *SERPINH1* gene, which encodes a chaperone protein (HSP47) necessary for fibrillar collagen sysnthesis was previously shown to be associated with PPROM [21].

The primary hypothesis explored in this study is that PPROM is the result of rare mutations or variants in the fetal genes involved in the elaboration of the amnion ECM. An initial Whole Exome Sequencing (WES) was performed, followed up by genotyping of select variants in additional samples of neonatal DNA from normal term pregnancies (controls) and pregnancies complicated by PPROM (cases). All of the neonates were delivered from mothers of self-reported African-American ancestry. In an effort to identify functional variants with large effect, the analysis was selectively focused on damaging mutations: either non-sense mutations or frameshift mutations that precluded production of a functional protein, and missense mutations, which were predicted to be damaging or potentially damaging. Furthermore, the investigation was restricted to genes whose disruption could theoretically promote PPROM by weakening the fetal membranes by disruption of the ECM fibrillar collagens and genes encoding proteins involved in their production.

We discovered that rare heterozygous, nonsense, frameshift and damaging missense mutations were more prevalent in the genomes of neonates born of pregnancies complicated by PPROM as compared to normal term controls. The combined burden of the rare damaging variants identified in the fetal genome yielded a statistically significant genetic association with PPROM. These results suggest that PPROM may be caused by infrequent genetic variants that modulate fetal membrane strength leading to weakening of the membranes and ultimately ending in premature rupture.

## Results

### Study population

The characteristics of the subjects used in the study are presented in Tables 1 and 2. There were no significant differences in maternal age, gravidity and parity between the cases and controls. As expected, the pregnancies complicated by PPROM had a significantly shorter gestational age at delivery than the term pregnancy control group (p < 0.001) and the PPROM neonatal birth weights were also significantly lower (p < 0.001) for both sample sets. Individual neonatal genetic ancestry estimates were calculated and used to compare the ancestry proportions between cases and controls and no differences were found. (Tables A and B in S1 File)

**Table 1:** Characteristics of the study population in the initial WES set.

Characteristic	Cases Mean (SD)	Controls Mean (SD)	p-value
Maternal Age (years)	27.57 (5.67)	25.8 (4.86)	0.198
Gestational Age at Delivery (weeks)	29.59 (4.17)	38.55 (1.19)	**< 0.001**
Neonatal Weight (kgs)	1.47 (0.65)	3.21 (0.53)	**< 0.001**
Gravidity	1.73 (0.54)	1.78 (0.48)	0.685
Parity	0.85 (0.72)	1.04 (0.62)	0.280

Cases (n = 49) and Controls (n = 20) were compared for key patient characteristics. Values represent means with SDs reported in parentheses for each group.

**Table 2:** Characteristics of the study population used for follow-up genotyping.

Characteristic	Cases Mean (SD)	Controls Mean (SD)	p-value
Maternal Age (years)	26.76 (5.91)	26.6 (6.02)	0.798
Gestational Age at Delivery (weeks)	31.05 (3.63)	39.47 (0.10)	**< 0.001**
Neonatal Weight (kgs)	1.7 (1.07)	3.35 (0.31)	**< 0.001**
Gravidity	1.91 (0.59)	1.89 (0.58)	0.09
Parity	1.06 (0.77)	1.10 (0.69)	0.62

Cases (n = 188) and Controls (n = 175) were compared for key patient characteristics. Values represent means with SDs reported in parentheses for each group.

#### Variant discovery

The analysis of WES was focused on damaging mutations (non-sense and frameshift mutations) and predicted damaging missense variants as identified by SIFT and Polyphen2 in the preselected genes of interest in neonatal DNA derived from of 49 pregnancies complicated by PPROM and 20 normal term pregnancies.

#### Variants identified in genes involved in ECM components and ECM Synthesis

The variants identified in the selected genes of interest (Refer to the Methods section for the list of genes investigated) in the initial WES are described in Table 3. The position-specific annotation of the variant in the protein (“within feature” column) describes the molecular processing outcome of that particular region in the final protein product. An additional 188 cases and 175 controls were genotyped for these variants.

**Table 3:** Variants identified in genes involved in ECM composition and synthesis.

Gene	SNP ID	Location	Allele Change	Type	AA Position(Residue Change)	Within Feature
BMP1	rs116360985	Chr8: 22058684	C > T	Nonsense	721 (R > Ter)	Chain
FKBP10	rs137853883	Chr17: 39975558	- > C	Frameshift	278 (G > R)	Chain
COL1A2	rs139528613	Chr7: 94028386	G > A	Missense	41 (R > H)	Propeptide
COL1A2	rs145693444	Chr7: 94038721	G > T	Missense	294 (V > F)	Chain
COL2A1	rs201234519	Chr12: 48391685	G > T	Missense	64 (P > H)	Propeptide
COL2A1	rs78690642	Chr12: 48367327	C > T	Missense	1374 (G > S)	Chain
COL5A1	rs116003670	Chr9: 137582842	G > A	Missense	65 (R > Q)	Chain
COL5A1	rs2229817	Chr9: 137726950	C > T	Missense	1757 (T > M)	Propeptide
COL5A1	rs61739195	Chr9: 137708884	C > T	Missense	1379 (P > S)	Chain

The positional and putative functional impact of the variants identified in the initial WES in the selected genes of interest and selected for additional genotyping are shown. (Propeptide = part of a protein that is cleaved during maturation or activation, chain = extent of polypeptide chain in the mature protein).

In the discovery WES, all variants identified were unique to cases. A heterozygous nonsense mutation in *BMP1* (rs116360985), an enzyme involved in procollagen processing, was discovered in 1 PPROM case. This mutation truncates the protein at amino acid residue 721 in a protein that has an isoform of 730 amino acids, so the functional significance is not clear. A heterozygous frameshift mutation (rs137853883) in the *FKBP10* gene, which encodes for a chaperone protein involved in ECM metabolism, was identified in 1 case. Heterozygous predicted damaging (Polyphen2 and SIFT) missense variants were found in *COL1A2*; rs139528613 in 3 cases and rs145693444 in 2 PPROM cases. Predicted damaging heterozygous missense variants were found in *COL5A1* (rs2229817, rs116003670 and rs61739195) in 3 different PPROM cases. Although rs61739195 and rs2229817 are predicted to be damaging by both Polyphen 2 and SIFT, ClinVar lists them as benign. All other mutations and missense variants are listed as having unknown clinical significance.

Interestingly, two missense variants, rs201234519 and rs78690642, both predicted to be damaging by Polyphen2 and SIFT were identified in *COL2A1*, which encodes a fibrillar collagen found in cartilage and tendons, and not previously thought to be expressed in amnion. rs78690642 was present in 5 cases and rs201234519 was present in one different case with none of the controls having either of the two variants. We performed an RT-PCR on RNA extracted from amnion tissue sample from normal term pregnancy (gestational age > 37 weeks, n = 1) using *COL2A1* specific primers. (S1 Fig). Thus, it is possible that *COL2A1* mRNA, and possibly protein, is expressed in amnion, and this has been overlooked in previous studies.

None of the heterozygous variants in the *COL1A2*, *COL2A1 and COL5A1* genes appeared in the same subject. However, one case had a predicted damaging mutation in *COL1A2* (rs139528613) as well as a nonsense mutation in *BMP1* (rs116360985).

Missense variants that were not predicted to be damaging using our stringent criteria were found in the fibrillar collagen genes as well as genes involved in their synthesis (Table C in S1 File). A number of these variants were novel and only detected in cases. In some instances one of the the predictive algorithms suggested that the variant was potentially damaging (e.g., rs201944190), but their significance with respect to PPROM remains to be evaluated.

Observed allele frequencies for the putative risk allele (RAF) of variants listed in Table 3 in the general populations ancestries as reported in the 1000 Genomes Project [22], the initial WES, as well as the follow up study are shown in Table 4. In total, WES identified 7 predicted to be damaging missense variants in the candidate genes involved in ECM formation in 14 cases, one frameshift variant in *FKBP10* and one nonsense mutation in *BMP1* in one case each and none in the 20 controls. In addition, the follow-up study revealed an increased burden of the damaging missense variants in 10 additional cases and 6 controls. The nonsense mutation in *BMP1* was identified in an additional 4 cases and 5 controls and the frameshift variant in *FKBP10* was not found in any of the additional samples genotyped. Combining the two sample sets revealed an increased frequency of the risk allele in cases as compared to controls.

**Table 4:** Allele frequencies of the variants identified.

SNP ID	RAF in CEU/AFR/ASW (1000 genomes)	RAF in Initial WES Case/ Control	RAF in Follow-Up Case/ Control	RAF in Combined Case/ Control
rs116360985	0.000/0.005/ 0.000	0.01/ 0	0.011/ 0.014	0.011/ 0.013
rs137853883	NA	0.01/ 0	0/ 0	0.002 / 0.00
rs139528613	0.000/0.011/ 0.025	0.031/ 0	0.006/ 0.009	0.011/0.008
rs145693444	0.000/0.004/ 0.008	0.02/ 0	0/ 0	0.004/ 0
rs201234519	0.000/0.002/ 0.000	0.01/ 0	0.006/ 0	0.006/ 0
rs78690642	0.000/0.014/ 0.025	0.05/ 0	0.009/ 0.003	0.018/ 0.003
rs116003670	0.000/0.007/ 0.000	0.01/ 0	0.006/ 0.006	0.006/ 0.005
rs2229817	0.000 /0.001/ 0.008	0.01/ 0	0.003/ 0	0.004/ 0
rs61739195	0.005 /0.000/ 0.000	0.01/ 0	0/ 0	0.002/ 0

The table shows the allele frequencies of the putative risk allele (RAF) of variants listed in Table 3 in the general populations of CEU—Northern Europeans from Utah (European-American), AFR–African (combined African populations) and ASW–Americans of African ancestry in Southwest USA (admixed African Americans) ancestries as reported in the 1000 Genomes Project and their observed risk allele frequencies in the initial WES and in the follow up study on an independent sample cohort used for custom genotyping, separate and combined. Please note that the AFR allele frequencies constitute a super population which includes the allele frequencies from all African populations in the 1000 Genomes Project including the ASW.

### Genetic association analysis

To determine if the rare variants collectively contributed to PPROM risk, we performed a genetic burden test using the combined initial WES and follow up genotypes including adjustment for West African ancestry. The omnibus SKAT-O test yielded a significant association for the rare variants with PPROM (p-value = 0.0225).

The genetic burden analysis tested genes that are involved in fibrillar collagen synthesis, many of which are known to be affected in the Ehlers-Danlos syndrome. Not included in our analysis were other genes that contribute to Ehlers-Danlos syndrome, which are not directly related to fibrillar collagens, including *TNXB*, which encodes for tenascin X, an ECM glycoprotein involved in matrix maturation and wound healing [17, 23]. Mutations in TNXB are associated with the hypermobility type of Ehlers-Danlos syndrome. Our initial WES identified novel heterozygous frameshift mutations in two PPROM cases and none in controls. In addition, several predicted to be damaging heterozygous missense mutations unique to cases were also identified. (Tables D and E in S1 File)

## Discussion

The present study tested the hypothesis that rare mutations and damaging variants in candidate genes involved in ECM production confer risk of PPROM-related preterm birth by altering fetal membrane integrity, resulting in weaker fetal membranes. We employed WES to survey 16 candidate genes in neonatal DNA from PPROM cases, and a smaller number of term pregnancy controls. This strategy identified rare mutations in an initial discovery sample and predicted damaging variants in PPROM cases that were not found in term pregnancy controls in the larger test sample. However, it should be noted that a larger number of mutations/damaging variants might have been detected had more cases and control DNA samples been subjected to WES. The relatively small sample size for mutation/damaging variant discovery is a limitation of this study, but the positive findings should encourage expanding this strategy to identify additional of rare mutation/variants. While these rare mutations/damaging variants themselves have low public health significance, our findings support a mechanistic hypothesis that could achieve clinical utility if in the future it would be possible to screen for the entire repertoire of rare variants.

Crosslinked networks of several collagen types constitute the major components of the ECM of the fetal membranes. Altered expression patterns or altered metabolism of any of the ECM or collagen components could lead to a loss of integrity of the fetal membranes [16–17]. Some studies have shown that PPROM membranes have an altered amnion collagen content as compared to normal term pregnancies [24–26]. We anticipated identifying variants that would disrupt the ECM, and looked for mutations and variants in genes encoding proteins involved in the production of the major ECM proteins in fetal membranes, especially variants that would alter the collagen content or structure of the amnion. With the exception of *BMP1* and *FKBP10*, we discovered no damaging mutations (nonsense, frameshift, splice junctions) in the selected genes of interest. *FKBP10* codes for a chaperone protein FKBP65 which is known to be associated with the extracellular matrix protein tropoelastin. Mutations in *FKBP10* have been found in several family members with osteogenesis imperfecta and clinical consequences of these mutations, one of which is the frameshift mutation identified in our study (rs137853883), included loss of FKBP65 protein function leading to delayed type I procollagen secretion and improper crosslinking of collagen [27, 28].

The tensile strength of fetal membranes is determined by the assembly of fibrillar collagens (I, III, V) into fibrils. The size of the fibrils is mainly determined by type V collagen in association with types I and III collagens and proteoglycans [16, 29]. Both *COL5A1* and *COL5A2* are involved in the production of type V collagen, which participates in early fibril initiation, determination of fibril structure and matrix organization [30, 31]. Type I collagen is involved in fibril formation and consists of two alpha-1 chains (*COL1A1*) and one alpha-2 chain (*COL1A2*). Mutations in the *COL1A1* and *COL1A2* genes are known to cause rare forms of Ehler’s Danlos Syndromes, types VIIA and B and osteogenesis imperfecta types I and II [17, 32, 33]. Mutations in *COL5A1* and *COL5A2* genes resulting in haploinsufficiency or structural modifications of type V collagen are common causes for classical Ehlers-Danlos Syndrome (types I and II) [34]. These disorders have been associated with increased risk of PPROM when the fetus/neonate is affected [17, 18, 20]. A case-control study with case-parent triads and case-mother dyads suggested a significant association of *COL5A1* (combined fetal-maternal association) and *COL5A2* (fetal association) with spontaneous preterm birth [30]. Our study identified several predicted to be damaging missense variants in the *COL1A2* and *COL5A1* genes that were unique to only cases in the initial WES. Missense variants were identified in the *COL1A1*, *COL3A1* and *COL5A2* genes but their predicted impact on protein function was benign.

Potentially damaging missense variants in the *COL2A1* gene were also discovered with increased frequency in PPROM cases. *COL2A1* codes for cartilage collagen and until now there has been no evidence of *COL2A1* expression in the amnion making the significance of this finding uncertain [35]. However, human amniotic membranes (HAM) have been used as a source of stem cells for chondrocyte culture, where chondrocytes grown on the chorionic side of the HAM express type II Collagen [36–37]. RT-PCR using mRNA from amnion tissue obtained from normal term pregnancy and *COL2A1* specific primers, revealed detectable *COL2A1* mRNA expression, raising the possibility of low levels of expression of *COL2A1* protein that might play a significant role in fetal membrane integrity. Alternatively, the detected RNA could be generated from illegitimate transcription of the *COL2A1* gene. The putative functional significance of the variants could be due to variants in genes that are in likage disequilibrium with the *COL2A1* SNPs or they could have a disrupting impact on overlapping coding sequences on the DNA strand opposite the *COL2A1* gene. A noncoding RNA (LOC105369752) of unknown function does reside there. There was no linkage disequilibrium (LD) information available for rs201234519. Rs78690642 is in LD with three other SNPs in the *COL2A1* gene (rs1455684563, rs76519927 and rs2071358).

It is important to note that there are significant disparities in the prevalence rates of PPROM with African-American women experiencing a 2-fold increased risk of PPROM as compared to European-American women. This disparity cannot be explained by socio-economic factors alone and genetic variation and gene-environment interactions are involved [8, 38]. Most of the rare variants described in this study are more prevalent in individuals of West African descent than European descent in the general population. Interestingly, this is also true for the *SERPINH1* promoter SNP that has previously been asscociated with PPROM [21]. Admixed populations such as the African Americans have varying proportions of West African and European genetic ancestry contributions across individuals and also differences in groups across different regions within the US [39–40]. Even though the sample set in our initial WES and the independent sample set on which custom genotyping was performed consist of individuals from two different regions, Richmond and Detroit respectively, the fact that their combined SKAT-O gives a significant association is promising, suggesting a higher functional impact of the rare variants identified. Moreover, the fact that the combined burden of rare variants, which are of African origin (all except rs61739195), is significantly associated with increased PPROM risk suggests that the increased prevalence of PPROM in African-American populations is partly attributed to these rare population-specific alleles.

There are potentially other rare variants inactivating transcription factors or disrupting transcription factor binding sites that might result in reduced production of fibrillar collagens in the amnion. These were not explored in our study, nor were epigenetic factors that could influence collagen gene expression. These should be explored in future research. Conversely, variants that affect expression of matrix degrading enzymes, particularly the matrix metalloproteinases, could contribute to PPROM risk. This has been suggested by association studies with promoter variants in the MMP1, MMP8, and MMP9 genes [41–43].

In summary, using a screen to detect deleterious genetic variants that could promote PPROM in pregnancies hosted by women of African-American descent, we discovered evidence that rare damaging non-sense and frameshift mutations and predicted to be damaging missense variants in a variety of genes involved in negatively modulating the ECM metabolism-related genes are more prevalent in neonates born from pregnancies complicated by PPROM than normal term pregnancies. Despite sample size being a limitation in our study, the variants identified strongly suggest that the fetal contribution to PPROM is polygenic and driven by multiple rare rather than common genetic variants.

## Methods

### Study population

The initial WES was performed on 49 case and 20 healthy term control neonatal DNA samples. Additional genotyping of select variants was performed on an independent cohort of 188 case and 175 control neonatal DNA samples. Subjects were self-reported African-American women and their neonates receiving obstetrical care at MCV Hospitals, Richmond, VA (all samples in the initial WES) and Hutzel Hospital in Detroit, MI. The study was approved by the Institutional Review Boards of MCV Hospitals, Richmond, VA (IRB Number: HM15009); Wayne State University (IRB Numbers: 103897MP2F (5R), 082403MP2F (5R), 110605MP4F, 103108MP2F, 052308MP2F) as well as NICHD (National Institute of Child Health and Human Development) (IRB Numbers: 0H97-CH-N065, OH98-CH-N001, OH97-CH-N067, OH99-CH-N056, OH09-CH-N014). Subjects from Hutzel Hospital, Detroit, MI were enrolled under both Wayne State University as well as NICHD protocols and thus respective IRB numbers for both institutes are provided. Written informed consent was obtained from mothers before sample collection. Demographic and clinical data were obtained from surveys and medical records. Control DNA samples (n = 20 + 175) were obtained from neonates of singleton pregnancies delivered at term (> 37 weeks of gestation) of mothers with no prior history of PPROM or preterm labor. Cases of PPROM (n = 49 + 188) were defined as neonates from pregnancies complicated by spontaneous rupture of membranes prior to 37 weeks of gestation. The diagnosis of membrane rupture was based on pooling of amniotic fluid in the vagina, amniotic fluid ferning patterns and a positive nitrazine test. Women with multiple gestations, fetal anomalies, trauma, connective tissue diseases and medical complications of pregnancy requiring induction of labor were excluded.

### Ancestry estimates

Genetic ancestry was estimated to control for the presence of population structure in all genetic association tests. Genetic ancestry estimates were generated in a two-way model of admixture, European and West African, for the neonates of each self-reported African American study subject using 102 ancestry informative markers (AIMs) single nucleotide polymorphisms with large allele frequency differences between ancestral populations. (Table F in S1 File) with mean allele frequency difference between ancestral populations delta (δ) = 0.733. The AIMs panel was derived from the overlap of the WES and the Illumina African American Admixture Mapping Panel (Illumina, San Diego, CA) and genotyped using a custom iPLEX assay (Agena Biosciences, San Diego, CA) for study subjects who were not part of the WES discovery set [44]. Prior allele frequencies derived from the HapMap West Africans (YRI, Yoruba in Ibadan, Nigeria) and Europeans (CEU, CEPH Utah residents with ancestry from northern and western Europe) were used to estimate individual genetic ancestry estimates following a maximum-likelihood approach [45–48]. Native American ancestry was not considered in the analysis since it is anticipated that, on average, there is a small contribution (<1%) of Native American ancestry in self-reported African Americans, especially those residing outside of the West and Southwest United States [49].

### Whole exome sequencing

Whole Exome Capture and Sequencing was performed on the initial set of samples at BGI (BGI, Cambridge, MA) using the SureSelect Target Enrichment System Capture Process and high-throughput sequencing on an Illumina HiSeq2000 platform with 50-100X coverage. Raw image files are processed by Illumina base calling Software 1.7 for base calling with default parameters and the sequences of each individual are generated as 90bp paired-end reads. The raw sequence data generated from the Illumina pipeline were used for bioinformatic analysis.

### Read mapping and pre-processing

Raw sequence data for each individual were mapped to the human reference genome (build hg19) using the BWA-MEM algorithm of Burrows-Wheeler Aligner (v 0.7.12) [50]. This was followed by a series of pre-processing steps–marking duplicates, realignment around indels and base quality recalibration. PCR duplicates were marked within the aligned reads using Picard tools. (http://picard.sourceforge.net) Next, mapping artifacts around indels were cleaned up using the RealignerTargetCreator, the IndelRealigner and the LeftAlignIndels walkers of the Genome Analysis ToolKit (GATK) [51, 52]. Inaccurate / biased base quality scores were recalibrated using the BaseRecalibrator, the AnalyzeCovariates and the PrintReads walkers of GATK, which use machine learning to model these errors empirically and adjust the quality scores accordingly. Alignment statistics for each sample were calculated on the “clean” sample BAM files.

### Variant discovery and quality filtering

The pre-processing steps were followed by variant calling, using the HaplotypeCaller walker of GATK on each sample BAM file. Variant sites are identified by taking into account the haplotype likelihood predicted by building Dr. Brujin-like graphs in regions where the data displays variation relative to the hg19 reference genome. This step is also guided using the dbSNP, and Mills and 1000G gold standard SNP and indel databases. The output is a set of unfiltered/raw SNP and indel calls in the Genomic Variant Call Format (gVCF) file. Sample-specific gVCFs were merged into a single VCF file and a cohort-wide joint genotyping was performed using the CombineGVCFs walker of GATK. Finally, Variant Quality Score Recalibration (VQSR) was performed to assign the statistical probability to each variant call and produce a call-set distilled to a desired level of truth sensitivity. The raw SNP call-set was filtered using the GATK VariantFilter module, with variants required to pass the following criteria–“QUAL > = 30” AND “DP > = 25”. The raw indel call-set was filtered with variants required to pass the following criteria–“QD > 2.0” AND “FS < 200.0” AND “InbreedingCoeff > -0.8” AND “ReadPosRankSum > -20.0".

### Annotation and filtering for genes and variants of interest

SnpEff was used for annotating the functional effects of *high-quality* SNPs and INDELs on genes, transcripts and protein sequences including: a) their genomic location (*i*.*e*., intron, 5’ or 3’ untranslated region, upstream/downstream of a transcript, or intergenic region); b) their consequence on protein sequence (*i*.*e*., stop-gained, missense, frameshift); c) known variants from dbSNP [53], ClinVar [54], and the 1000 Genomes Project [22].

A total of sixteen candidate genes were selected for investigation of rare variants (Table 5) based on their involvement in the extra-cellular matrix (ECM) composition and synthesis and previously linked to connective tissue disorders such as classical types of Ehlers-Danlos syndrome (types I and II) as well as Ehler’s-Danlos Syndrome types VIIA and VIIB, osteogenesis imperfecta type II and restrictive dermopathy [17]. These genes encode for major ECM components including fibrillar collagens (*COL1A1*, *COL1A2*, *COL2A1*, *COL3A1*, *COL5A1*, *COL5A2)* and associated proteins *(CRTAP*, *ELN*) as well as enzymes involved in collagen processing and ECM production (*ADAMTS2*, *BMP1*, *LEPRE1*, *LOX*, *LOXL1*, *SERPINH1*, *ZMPSTE24*, *FKBP10*). Further analyses were focused on variants affecting only coding regions of the selected genes to best identify functional variation and this included nonsense, frameshift, splice site and damaging missense variants. Damaging missense variants were selected on the basis of most deleterious predictions in both Polyphen2 (HumDiv—probably damaging) as well as SIFT (damaging) platforms.

**Table 5:** Candidate genes selected for analysis.

Category	Gene IDs
Fibrillar collagens	*COL1A1*, *COL1A2*, *COL2A1*, *COL3A1*, *COL5A1*, *COL5A2*
Cartilage & Collagen associated proteins	*CRTAP*, *ELN*
Enzymes/ Proteins involved in collagen processing and ECM synthesis	*ADAMTS2*, *BMP1*, *LEPRE1*, *LOX*, *LOXL1*, *SERPINH1*, *ZMPSTE24*, *FKBP10*

The 16 genes selected for investigation of rare damaging variants are listed and categorized based on their roles in ECM composition and synthesis.

### Custom genotyping

The variants identified and selected for further analysis from Whole Exome Sequencing (Table 3) were validated and additional samples (an independent cohort of additional 188 cases and 175 controls) were genotyped for the selected variants. Genotyping was performed on the Agena (previously Sequenom) MassArray iPLEX platform following manufacturer’s instructions [55] at the University of Minnesota Genomics Center.

### Testing for genetic association

The combined set of variants identified in the initial WES and by additional genotyping were tested for genetic association using the combined Optimized Sequence Kernel Association Test (SKAT-O) software package in R version 3.2.3 [56–58] with default parameters adjusting for small sample size and ancestry estimates as covariates. A burden test was selected because the variants under study were rare (variant frequency in sample set < the calculated fixed frequency threshold *T* = 0.034), in the coding region and known/ expected to alter amino acid sequence and thus assumed to contribute to risk with same direction of effect [59]. Given the extremely low minor allele frequencies for the mutations and damaging variants, very large sample sizes on the order of several thousand cases and controls would be needed to rule out significant associations for univariate tests of individual mutation/damaging variants and PPROM.

### Statistical analysis

Mean levels of demographic variables were tested using a 2-tailed Student’s t-test. Count data (for gravidity and parity) was square-root transformed before performing tests. P-values < 0.05 were considered statistically significant.

## Supporting information

S1 Fig COL2A1 mRNA expression in amnion sample from normal term pregnancy. RT-PCR gel image for *COL2A1* (100 bp) using 1 μg RNA from amnion tissue sample (lane 3) obtained from normal term pregnancy (gestational age > 37 weeks) showing mRNA expression of the *COL2A1* gene in the amnion. RNA from primary cultures of human ear chondrocytes (provided by Dr. Barbara Boyan, Virginia Commonwealth University) was used as a positive control for *COL2A1* gene expression (lane 2). The *COL2A1* cDNA amplicon was cloned and sequence—verified. (*COL2A1* Primers: 5- CTCGCGGTGAACCTGGTACT-3 and 5- GCACCAGCAGATCCTTTGGC-3).
(PDF)

S1 File Comparison of neonatal ancestry between cases and controls in the initial WES. Neonatal case (n = 49) and control (n = 20) samples were compared for European and West-African ancestry proportions. Values represent mean genetic ancestry estimates generated using two-way model of admixture following maximum likelihood method with SD in parentheses **(Table A)**. **Comparison of neonatal ancestry between cases and controls in the follow-up genotyping study.** Neonatal case (n = 188) and control (n = 175) samples were compared for European and West-African ancestry proportions. Values represent mean genetic ancestry estimates generated using two-way model of admixture following maximum likelihood method with SD in parentheses **(Table B). Missense variants not selected.** Missense variants identified by WES in the genes of interest but not selected for analysis in this study are listed along with their SIFT and Polyphen2 predictions and the observed putative risk allele frequencies (RAF) for cases (n = 49) and controls (n = 20). Novel variants were submitted to dbSNP and ss ids are provided. In cases of multiple SIFT and PolyPhen2 predictions for variants that were annotated to multiple isoforms, only distinct predictions are listed (i.e. does not reflect the total number of isoforms that were actually annotated). (SIFT predictions: T = Tolerated, D = Damaging; Polyphen2 predictions: B = Benign, P = Possibly Damaging, D = Probably Damaging) **(Table C). Variants identified in the *TNXB* gene.** The positional and putative functional impact of the variants identified in the initial WES in the *TNXB* gene are shown. All positional information corresponds to the full length precursor Tenascin-X protein of 4242 amino acids annotating to transcript NM_019105. (chain = extent of polypeptide chain in the mature protein) **(Table D). Allele frequencies of the variants identified in the *TNXB* gene.** The table shows the allele frequencies of the putative risk allele (RAF) of variants listed in S5 Table in the general populations of CEU—Northern Europeans from Utah (European American), AFR–African (combined African populations) and ASW–Americans of African ancestry in Southwest USA (admixed African Americans) ancestries as reported in the 1000 Genomes Project [22] and their observed risk allele frequencies in the initial WES. Please note that the AFR allele frequencies constitute a super population, which includes the allele frequencies from all African populations in the 1000 Genomes Project including the ASW **(Table E). Ancestry Informative Markers (AIMs) used for calculation of ancestry estimates** The 102 SNPs listed above were used as ancestry informative markers (AIMs) to calculate genetic ancestry estimates. The mean allele frequency difference between the ancestral populations (West African and European) used to generate estimates was δ = 0.733 **(Table F)**.
(DOCX)
