# Supplementary figures and images for: Rare mutations and potentially damaging missense variants in genes encoding fibrillar collagens and proteins involved in their production are candidates for risk for preterm premature rupture of membranes
Source: PLoS One. 2017 Mar 27;12(3):e0174356. doi: 10.1371/journal.pone.0174356 (PMC5367779; doi:10.1371/journal.pone.0174356)

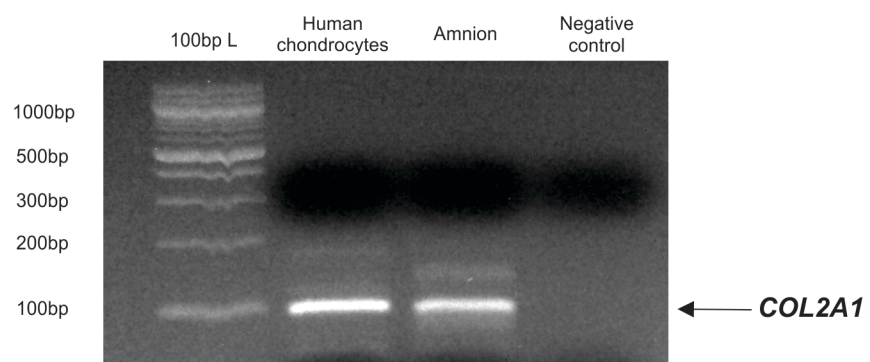

Supplement: S1 Fig — RT-PCR gel image for COL2A1 (100 bp) using 1 μg RNA from amnion tissue sample (lane 3) obtained from normal term pregnancy (gestational age > 37 weeks) showing mRNA expression of the COL2A1 gene in the amnion. RNA from primary cultures of human ear chondrocytes (provided by Dr. Barbara Boyan, Virginia Commonwealth University) was used as a positive control for COL2A1 gene expression (lane 2). The COL2A1 cDNA amplicon was cloned and sequence—verified. (COL2A1 Primers: 5- CTCGCGGTGAACCTGGTACT-3 and 5- GCACCAGCAGATCCTTTGGC-3). (PDF) [file pone.0174356.s001.pdf]
