# Supplementary material for: Rare mutations and potentially damaging missense variants in genes encoding fibrillar collagens and proteins involved in their production are candidates for risk for preterm premature rupture of membranes
Source: PLoS One. 2017 Mar 27;12(3):e0174356. doi: 10.1371/journal.pone.0174356 (PMC5367779; doi:10.1371/journal.pone.0174356)
Supplement: S1 File — Neonatal case (n = 49) and control (n = 20) samples were compared for European and West-African ancestry proportions. Values represent mean genetic ancestry estimates generated using two-way model of admixture following maximum likelihood method with SD in parentheses (Table A). Comparison of neonatal ancestry between cases and controls in the follow-up genotyping study. Neonatal case (n = 188) and control (n = 175) samples were compared for European and West-African ancestry proportions. Values represent mean genetic ancestry estimates generated using two-way model of admixture following maximum likelihood method with SD in parentheses (Table B). Missense variants not selected. Missense variants identified by WES in the genes of interest but not selected for analysis in this study are listed along with their SIFT and Polyphen2 predictions and the observed putative risk allele frequencies (RAF) for cases (n = 49) and controls (n = 20). Novel variants were submitted to dbSNP and ss ids are provided. In cases of multiple SIFT and PolyPhen2 predictions for variants that were annotated to multiple isoforms, only distinct predictions are listed (i.e. does not reflect the total number of isoforms that were actually annotated). (SIFT predictions: T = Tolerated, D = Damaging; Polyphen2 predictions: B = Benign, P = Possibly Damaging, D = Probably Damaging) (Table C). Variants identified in the TNXB gene. The positional and putative functional impact of the variants identified in the initial WES in the TNXB gene are shown. All positional information corresponds to the full length precursor Tenascin-X protein of 4242 amino acids annotating to transcript NM_019105. (chain = extent of polypeptide chain in the mature protein) (Table D). Allele frequencies of the variants identified in the TNXB gene. The table shows the allele frequencies of the putative risk allele (RAF) of variants listed in S5 Table in the general populations of CEU—Northern Europeans from Utah (European Americ [file pone.0174356.s002.docx]

**S1 File**

**Table A. Comparison of neonatal ancestry between cases and controls in the initial WES:**

| **Ancestry** | **Cases**  **Mean (SD)** | **Control**  **Mean (SD)** | **p-value** |
| --- | --- | --- | --- |
| European | 0.295 (0.070) | 0.314 (0.107) | 0.463 |
| West-African | 0.705 (0.070) | 0.685 (0.107) | 0.463 |

Neonatal case (n = 49) and control (n = 20) samples were compared for European and West-African ancestry proportions. Values represent mean genetic ancestry estimates generated using two-way model of admixture following maximum likelihood method with SD in parentheses.

**Table B. Comparison of neonatal ancestry between cases and controls in the follow-up genotyping study.**

| **Ancestry** | **Cases**  **Mean (SD)** | **Control**  **Mean (SD)** | **p-value** |
| --- | --- | --- | --- |
| European | 0.1823 (0.083) | 0.1658 (0.119) | 0.134 |
| West-African | 0.8176 (0.083) | 0.8341 (0.119) | 0.134 |

Neonatal case (n = 188) and control (n = 175) samples were compared for European and West-African ancestry proportions. Values represent mean genetic ancestry estimates generated using two-way model of admixture following maximum likelihood method with SD in parentheses.

**Table C. Missense variants not selected.**

| Gene | SNP ID | Location | Allele Change | SIFT prediction | PolyPhen2_HDIV prediction | RAF  (Cases) | RAF  (Controls) |
| --- | --- | --- | --- | --- | --- | --- | --- |
| COL1A1 | rs142570406 | chr17: 48264244 | G > C | T | B | 0.01 | 0 |
| COL1A1 | rs116794104 | chr17: 48272827 | C > T | T | B | 0.01 | 0.025 |
| COL1A1 | rs1800215 | chr17: 48265495 | T > C | T | B | 0.93 | 1 |
| COL1A1 | rs145446512 | chr17: 48266851 | C > T | T | B | 0 | 0.025 |
| COL1A2 | ss2019492554 | chr7: 94029523 | C > T | T | P,P | 0 | 0.025 |
| COL1A2 | rs42524 | chr7: 94043239 | C > G | T | B | 0.91 | 0.85 |
| COL1A2 | rs150124840 | chr7: 94057691 | C > T | T | P | 0.01 | 0 |
| COL1A2 | rs35820023 | chr7: 94056353 | G > A | T | P | 0 | 0.025 |
| COL2A1 | rs140368756 | chr12: 48372444 | G > A | T | B,B | 0 | 0.025 |
| COL2A1 | rs3803183 | chr12: 48398080 | T > A | T | B,B | 0.56 | 0.575 |
| COL2A1 | rs34392760 | chr12: 48391657 | T > A | T | B,B | 0.01 | 0.025 |
| COL2A1 | rs12721427 | chr12: 48368541 | C > T | T | B,B | 0.03 | 0 |
| COL2A1 | rs2070739 | chr12: 48367976 | C > T | T | D,D | 0.05 | 0.075 |
| COL2A1 | rs201823490 | chr12: 48380213 | G > A | T | B,B | 0.03 | 0 |
| COL3A1 | rs201220788 | chr2: 189870163 | G > A | T | D | 0 | 0.025 |
| COL3A1 | rs373838193 | chr2: 189862102 | C > T | T | D | 1 | 0 |
| COL3A1 | rs1800255 | chr2: 189864080 | G > A | T | B | 0.1 | 0.1 |
| COL3A1 | rs1801183 | chr2: 189863424 | C > T | T | P | 0.01 | 0 |
| COL3A1 | rs41263775 | chr2: 189864044 | C > G | T | B | 0.01 | 0 |
| COL3A1 | rs1516446 | chr2: 189875421 | T > G | T | B | 1 | 1 |
| COL5A1 | rs147008954 | chr9: 137593122 | C > G | D | P | 0.01 | 0 |
| COL5A1 | ss2019492556 | chr9: 137698068 | G > A | T | D | 0 | 0.025 |
| COL5A1 | rs61729495 | chr9: 137734039 | G > A | T | B | 0.01 | 0 |
| COL5A1 | rs61735045 | chr9: 137642654 | G > A | T | D | 0.01 | 0 |
| COL5A1 | ss2019492555 | chr9: 137620639 | A > T | T | B | 0 | 0.025 |
| COL5A2 | rs35852101 | chr2: 189931144 | A > G | N/A | B,P | 0.01 | 0 |
| COL5A2 | rs6434313 | chr2: 189916110 | C > G | N/A | B | 0.01 | 0.025 |
| CRTAP | rs145623565 | chr3: 33161975 | A > C | T,T | P | 0.01 | 0 |
| CRTAP | ss2019492550 | chr3: 33155942 | G > C | T | D | 0.01 | 0 |
| ELN | ss2019492553 | chr7: 73474329 | G > T | T | B | 0.01 | 0 |
| ELN | rs2071307 | chr7: 73470714 | G > A | T | B | 0.21 | 0.25 |
| ELN | rs61734581 | chr7: 73459575 | G > T | T | D | 0.02 | 0.05 |
| ELN | rs144026807 | chr7: 73474485 | C > T | T | B | 0.01 | 0 |
| ELN | rs137987089 | chr7: 73474484 | G > T | T | P | 0.01 | 0 |
| ELN | rs140425210 | chr7: 73477524 | G > A | T | D | 0.01 | 0.025 |
| ELN | ss2019492552 | chr7: 73457326 | G > A | T | D,P | 0.01 | 0 |
| ELN | rs145612009 | chr7: 73462008 | G > T | T | D | 0.01 | 0 |
| ELN | rs34945509 | chr7: 73472000 | A > G | T | P | 0 | 0.05 |
| ELN | rs144341345 | chr7: 73470729 | C > G | T | P | 0.03 | 0.025 |
| ADAMTS2 | rs35445112 | chr5: 178555097 | C > T | T | B | 0 | 0.05 |
| ADAMTS2 | rs398829 | chr5: 178634672 | C > T | T | B | 0.43 | 0.525 |
| ADAMTS2 | rs59567206 | chr5: 178634704 | T > C | T | B | 0.08 | 0.075 |
| ADAMTS2 | rs11750821 | chr5: 178634683 | C > T | T | B | 0.01 | 0.075 |
| ADAMTS2 | rs143764421 | chr5: 178634657 | C > T | T | B | 0 | 0.025 |
| ADAMTS2 | rs35372714 | chr5: 178563002 | C > T | T | B | 0 | 0.025 |
| ADAMTS2 | rs1054480 | chr5: 178540975 | G > A | T | P | 0.07 | 0.1 |
| ADAMTS2 | rs79330641 | chr5: 178549719 | G > A | T | B | 0.02 | 0.05 |
| ADAMTS2 | ss2019492551 | chr5: 178771124 | C > T | T | D,P | 0.01 | 0 |
| SERPINH1 | ss2019492557 | chr11: 75277973 | G > C | T | P | 0 | 0.025 |
| SERPINH1 | rs141721173 | chr11: 75277974 | C > A | T | B | 0 | 0.025 |
| SERPINH1 | rs138784081 | chr11: 75277959 | A > G | D | B,P | 0.01 | 0 |
| FKBP10 | rs34764749 | chr17: 39974642 | A > G | T | B | 0.12 | 0.1 |
| FKBP10 | rs76022961 | chr17: 39978052 | G > A | T | P,B | 0.02 | 0.05 |
| FKBP10 | rs201944190 | chr17: 39978604 | G > A | D | B | 0.01 | 0 |
| FKBP10 | rs145424241 | chr17:39969495 | A > G | T | D | 0.01 | 0 |

Missense variants identified by WES in the genes of interest but not selected for analysis in this study are listed along with their SIFT and Polyphen2 predictions and the observed putative risk allele frequencies (RAF) for cases (n = 49) and controls (n = 20). Novel variants were submitted to dbSNP and ss ids are provided. In cases of multiple SIFT and PolyPhen2 predictions for variants that were annotated to multiple isoforms, only distinct predictions are listed (i.e. does not reflect the total number of isoforms that were actually annotated). (SIFT predictions: T = Tolerated, D = Damaging; Polyphen2 predictions: B = Benign, P = Possibly Damaging, D = Probably Damaging).

**Table D. Variants identified in the *TNXB* gene**

| **SNP ID** | **Location** | **Allele Change** | **Type** | **AA Position**  **(Residue Change)** | **Within Feature** |
| --- | --- | --- | --- | --- | --- |
| ss2137463759 | Chr 6: 32011594 | T > C | Missense | 3819 (Y > C) | Chain |
| rs142627164 | Chr 6: 32012906 | C > T | Missense | 3600 (V > M) | Chain |
| rs121912575 | Chr 6: 32052313 | C > T | Missense | 1108 (V > M) | Chain |
| rs375984855 | Chr 6: 32064275 | C > T | Missense | 452 (C > Y) | Chain |
| ss2137463760 | Chr 6: 32037603 | G > A | Missense | 1772 (R > C) | Chain |
| ss2137463758 | Chr 6: 31978524 | TG > T | Frameshift | 3837 (Q > fs) | Chain |

The positional and putative functional impact of the variants identified in the initial WES in the *TNXB* gene are shown. All positional information corresponds to the full length precursor Tenascin-X protein of 4242 amino acids annotating to transcript NM_019105. (chain = extent of polypeptide chain in the mature protein).

**Table E. Allele frequencies of the variants identified in the *TNXB* gene.**

| **SNP ID** | **RAF in**  **CEU/AFR/ASW**  **(1000 genomes)** | **RAF in**  **Initial WES**  **Case/ Control** |
| --- | --- | --- |
| ss2137463759 | NA | 0.01/0.00 |
| rs142627164 | 0.00/0.019/0.033 | 0.01/0.00 |
| rs121912575 | 0.00/0.00/0.00 | 0.01/0.00 |
| rs375984855 | 0.00/0.002/0.00 | 0.01/0.00 |
| ss2137463760 | NA | 0.01/0.00 |
| ss2137463758 | NA | 0.02/0.00 |

The table shows the allele frequencies of the putative risk allele (RAF) of variants listed in Table D in the general populations of CEU - Northern Europeans from Utah (European-American), AFR –African (combined African populations) and ASW – Americans of African ancestry in Southwest USA (admixed African Americans) ancestries as reported in the 1000 Genomes Project [22] and their observed risk allele frequencies in the initial WES. Please note that the AFR allele frequencies constitute a super population, which includes the allele frequencies from all African populations in the 1000 Genomes Project including the ASW.

**Table F. Ancestry Informative Markers (AIMs) used for calculation of ancestry estimates**

| rs819980 | rs4667419 | rs2224391 | rs2293966 | rs7990216 | rs2593595 |
| --- | --- | --- | --- | --- | --- |
| rs3765337 | rs3213843 | rs2328893 | rs2236418 | rs17068920 | rs2874686 |
| rs1768560 | rs6434811 | rs195418 | rs2394931 | rs3861128 | rs14336 |
| rs12410334 | rs4596126 | rs6577 | rs10887651 | rs3211416 | rs3764962 |
| rs563921 | rs2197896 | rs56237735 | rs1056877 | rs927686 | rs10871774 |
| rs11164649 | rs1122818 | rs2286453 | rs7935419 | rs3825663 | rs2285972 |
| rs835574 | rs6549722 | rs1860521 | rs7947368 | rs1131877 | rs7254885 |
| rs2814778 | rs9288952 | rs28608208 | rs4148636 | rs12908877 | rs901792 |
| rs12065033 | rs641320 | rs17154865 | rs523200 | rs199138 | rs1205817 |
| rs1923949 | rs34443048 | rs6464211 | rs3740767 | rs7170666 | rs7255638 |
| rs291102 | rs7659 | rs4716656 | rs647756 | rs710079 | rs6132532 |
| rs291090 | rs2867697 | rs6558052 | rs647975 | rs7764 | rs6060262 |
| rs2666839 | rs3733778 | rs4733951 | rs899653 | rs7201030 | rs2151512 |
| rs1222158 | rs7735863 | rs10957952 | rs2242138 | rs2293067 | rs16998089 |
| rs11124663 | rs6879867 | rs10808483 | rs4767461 | rs2958475 | rs1009787 |
| rs1025104 | rs77937 | rs3764797 | rs1051431 | rs854655 | rs67153574 |
| rs1446585 | rs30533 | rs3849903 | rs11831366 | rs9891361 | rs932327 |

The 102 SNPs listed above were used as ancestry informative markers (AIMs) to calculate genetic ancestry estimates. The mean allele frequency difference between the ancestral populations (West African and European) used to generate estimates was δ = 0.733.
